# Leech Therapy in Nearly Total Amputation of Fingers Without Vascular Repair: A Case Report

**DOI:** 10.5812/ircmj.6897

**Published:** 2014-05-05

**Authors:** Mohammad TarazJamshidi, Farshid Bagheri, Masud Mirkazemi, Sara Amelfarzad, Hami Ashraf, Mehran Azami, Mohammad Taghi Peivandi

**Affiliations:** 1 Faculty of Medicine, Mashhad Orthopedic Research Center, Mashhad University of Medical Sciences, Mashhad, IR Iran; 2 Faculty of Pharmacy, Mashhad University of Medical Sciences, Mashhad, IR Iran; 3 Department of Research and Education, Razavi Hospital, Mashhad, IR Iran

**Keywords:** Leeches, Amputation, Vascular System Injuries, Hyperemia

## Abstract

**Introduction:**

In the absence of microvascular replantation or in crash injury cases in which obtaining an acceptable function is not possible, amputation of the injured finger seems to be the best treatment modality. Some studies recommended leech therapy for this kind of injury after vascular repair to decrease venous congestion.

**Case Presentation:**

In this case report, the authors presented a case of leech therapy after near total amputation of the fingers. A 25-year-old patient was admitted following a sawing injury with crashed bundles of the third, fourth and fifth fingers. Microvascular surgery was not performed because of crush injury.

**Discussion:**

After a simple repair and pin fixation, the patient was treated using leech therapy. The result was satisfactory. The third and fourth fingers were salvaged. It seems that in cases where a small part of the skin is still attached to the amputated part, even with complete crash of both bundles, leech therapy can help salvage the amputated fingers.

## 1. Introduction

Management of finger amputation, due its difficulties, is a dilemma and in many cases microvascular replantation is not possible, especially in those with crash injury. Since the purpose of replantation is obtaining a near normal function (motion, sensation, and handgrip), amputation of the injured finger seems to be the best treatment. Nowadays, microsurgical techniques have improved and more and more finger replantations are performed (1). However, replantation would be possible if there is the plausibility of microsurgery (patient’s condition, hospital facilities).

Venous congestion, even for a short period, could cause thrombosis at the digital arteriole anastomosis, and may result in replantation loss (2). Leech therapy has a long history in different branches of medicine (3). The use of leech in finger replantation after microvascular surgery has been reported to decrease venous congestion (4).

In this report, we presented a case in which microvascular surgery was not performed (due to crush injury) and after a simple repair, leeches therapy was performed. The idea behind this technique is to suck blood into small remaining vessels from the volar part of the finger (the only attached skin).

## 2. Case Presentation

**Figure 1.**
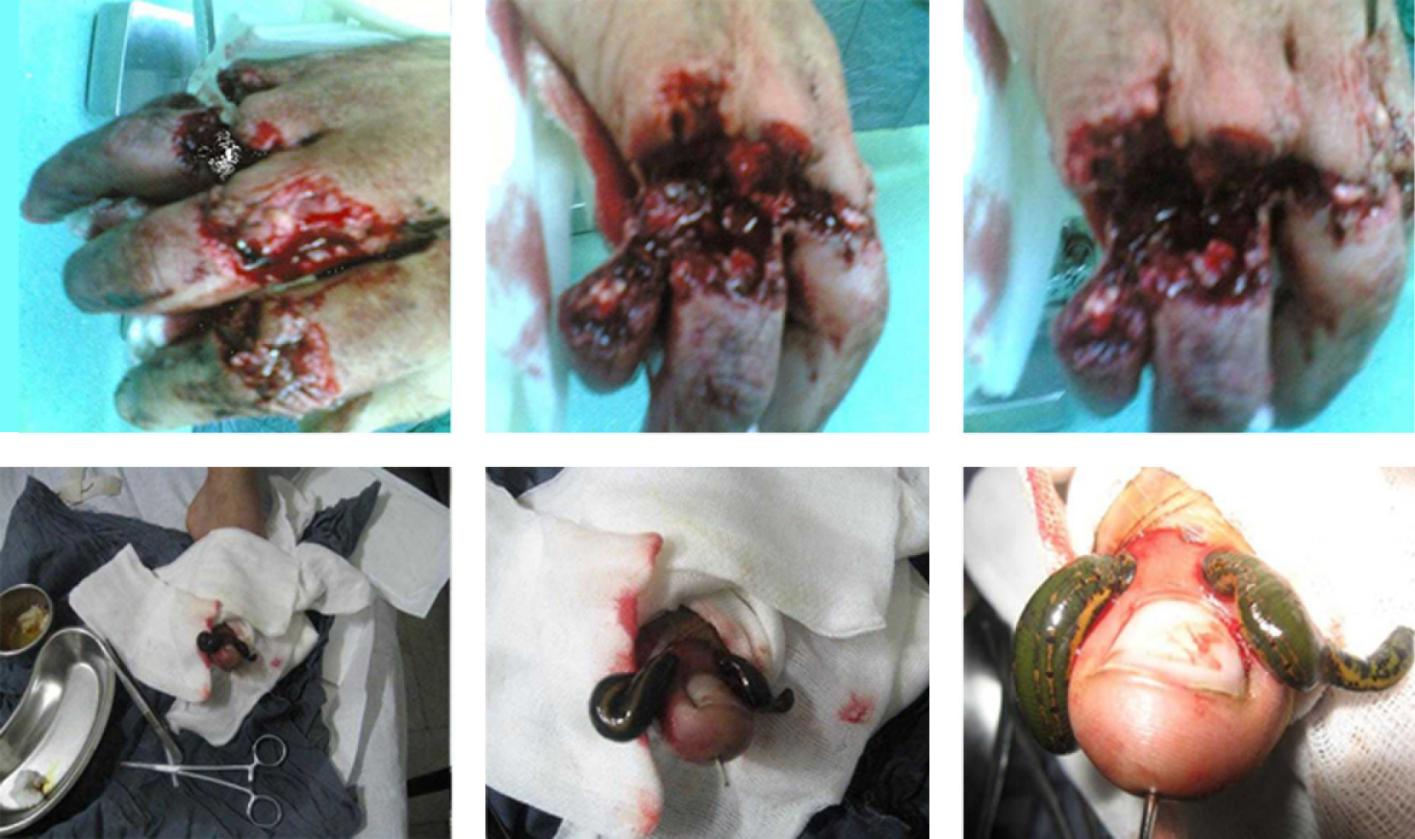
A, B, C. Amputated Fingers; Crush Injury of Neurovascular Bundles. D, E, F. Leech Therapy in Action. To Ensure Continuous Bleeding of the Repaired Fingers, the Leeches When Necessary Were Replaced by New Hungry Ones.

A 25-year-old man was admitted to the emergency ward of Shahid-Kamyab Hospital, Mashhad, Iran, with traumatic-near-amputation condition of the third, fourth and fifth fingers of the left hand in January 2011. Trauma occurred during an occupational accident where the carpenter’s saw injured his fingers in the proximal phalanx, as illustrated in figure one (Figure 1 A, 1B, 1C). The patient had no previous history of disease or surgery.

He was immediately transferred to the operation theatre. The lateral neurovascular bundle of the third finger was intact. Nevertheless, the third finger was completely ischemic and with no capillary filling. In fourth and fifth fingers, neurovascular components were crushed and only a small volar skin flap existed. According to the situation of hospital, having no access to microvascular surgery equipment, and vascular surgeon consultation, microvascular replantation was not applicable. After a meticulous debridement, bone was fixed using a K-wire. Soft tissue was sutured without vascular surgery. The cutaneous nerves of the fingers were not repaired. After reaching a good alignment, capillary filling of the third finger was not returned. It may be due to severe intimal lesion and thrombosis. The third finger could be assumed near amputated.

Appropriate prophylactic antibiotic and low molecular weight heparin commenced (40 mg, SC, BID). Leech therapy commenced 12 hours after the operation. The leeches applied were obtained from breeders, since they were not available in the center (Figure 1D, 1E) (3). Leeches were applied until they were satiated (mean 20 minutes) on a daily basis. In the beginning days, a small incision was applied to cause a minimal amount of bleeding to encourage leeches to bite the ischemic fingers. The leeches were handled by pancit. After each leech therapy, blood oozing continued for one hour, which it amount was less in early days, increasing day by day. In general, 15 leeches were used for this patient. On the first postoperative day, his fingers were assessed for color, capillary refill and blood oozing every two hours. Where necessary, leeches were applied to decrease venous congestion.

To ensure continuous bleeding of the repaired fingers, the leeches were observed and, where necessary, they were replaced by new hungry ones (Figure 1F). On the seventh day, the fifth repaired finger was lost, but the third and fourth fingers survived. There was no need to transfusion during and after the treatment by close monitoring of the patient.

**Figure 2.**
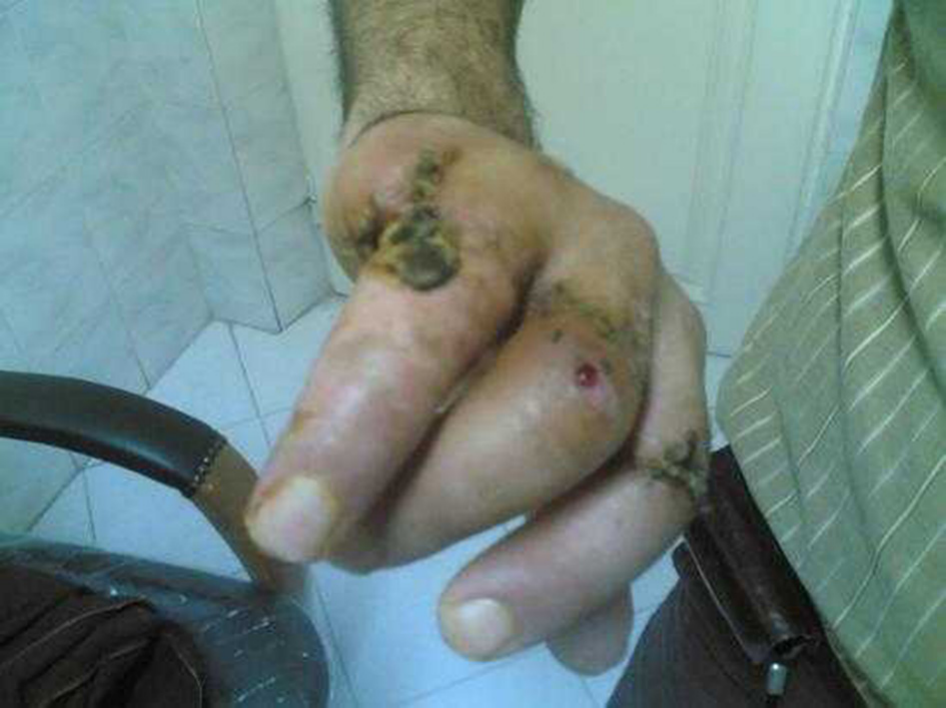
Range of Motions of Fingers, 2 Months After Surgery.

The patient left the hospital in the 13th day. He returned to his sophisticated work three months later with an active and functional left hand. Three months after surgery, the range of motion of the third and fourth fingers was 30 degrees and they both lacked skin sensation (Figure 2). Due to the novelty of method, the nurses were provided with necessary information and skills gathered through informants such as physicians, veterinarians, and leech breeders.

## 3. Discussion

Occupational finger amputation is one of common injuries, occurred in productive age of patients, (5, 6), especially in industry and agriculture workers (7). In many cases, microvascular surgery is not possible.

The plausible methods in the treatment of amputated fingers include microsurgical replantation and composite graft techniques (8). Because of anastomosing branches between vessels of the finger, the remaining intact skin flap may preserve the survival of distal part if we help decongestion of the part with leech therapy.

The US FDA has approved Leech therapy in microsurgeries and plastic surgeries. During the formation of collateral vessels, leeches help blood circulation and consequently decrease venous congestion (3). This is not a routine rule and no one denies the valuable role of vascular reconstruction whenever possible and available, but we have witnessed few patients with only a partial skin flap attachment in the injured site where the finger had been survived.

Tuncali et al. reported the excellent results of leech application on two cases with ring avulsion injuries where both had normal artery and lacerated vein (9). In another study, Shenfeld also reported promising results of this method in the treatment of venous insufficiency in a replanted digit (4).

What distinguishes this manuscript is the fact that leech therapy was performed without first performing a microvascular surgery due to the type of vascular damage. Although the amputated fingers are insensitive to pain, the leech bites are pain-free (10). This fact increased the patient’s acceptance to complete the treatment course.

Chen et al. proposed controlled bleeding from the fingertip with leech therapy in fingertip replantation (11). For this goal, various methods such as paraungual incision, and distal fish-mouth incision are used (12, 13). However, we believe that it is important to control the amount of bleeding to minimize blood loss and the possibly need for transfusion. It needs more study to confirm and find indications and contraindications.

The existence of hyaluronidase and a histamine-like vasodilator in the leech’s saliva are the main factors in blood pumping through small attached vessels of the skin (14). Bacterial flora in the leech gut is responsible for blood digestion. This normal flora could cause wound infections and bacteraemia in host when medicinal leeches are used (15).

Although the application of appropriate prophylactic antibiotic therapy is obvious, as in this case close consideration of the patient for probable sepsis was necessary. Levine et al., reported a case of *Aeromonas hydrophila *septicemia after leech therapy, which was resistant to standard prophylactic antibiotic therapy (16).

In our case, according to the severity of injury to the three fingers, they were at a very high risk of amputation. However, with the help of this technique, two of the three fingers survived. Still there is a need to nerve repair surgery in future. As it is shown in Figure 1, the amputation of the fifth finger was due the severity of injury, which was beyond the treatment. However, if a microvascular surgeon was available, it could be survived. In this study, the authors reported a case of treatment of near amputated fingers without microvascular surgery. The authors strongly advise leech therapy technique in cases in which microvascular surgery is impossible to increase the limb salvage chance.
